# Insulin Signaling in Arthritis

**DOI:** 10.3389/fimmu.2021.672519

**Published:** 2021-04-30

**Authors:** Cesare Tripolino, Jacopo Ciaffi, Valentina Pucino, Piero Ruscitti, Nina van Leeuwen, Claudio Borghi, Roberto Giacomelli, Riccardo Meliconi, Francesco Ursini

**Affiliations:** ^1^ Geriatric Medicine Unit, Department of Medical Functional Area, “San Giovanni di Dio” Hospital, Crotone, Italy; ^2^ Medicine and Rheumatology Unit, IRCCS Istituto Ortopedico Rizzoli (IOR), Bologna, Italy; ^3^ Institute of Inflammation and Ageing, University of Birmingham and Queen Elizabeth Hospital, Birmingham, United Kingdom; ^4^ Rheumatology Unit, Department of Biotechnological and Applied Clinical Sciences, University of L’Aquila, L’Aquila, Italy; ^5^ Rheumatology Department, Leiden University Medical Center, Leiden, Netherlands; ^6^ Unità Operativa Medicina Interna Cardiovascolare—IRCCS Azienda Ospedaliera-Universitaria, Bologna, Italy; ^7^ Rheumatology and Immunology Unit, Department of Medicine, University of Rome “Campus Biomedico”, Rome, Italy; ^8^ Department of Biomedical and Neuromotor Sciences (DIBINEM), Alma Mater Studiorum University of Bologna, Bologna, Italy

**Keywords:** rheumatoid arthritis, insulin, insulin receptor, metabolism, T cell, macrophage, synoviocyte

## Abstract

Inflammatory arthritis is burdened by an increased risk of metabolic disorders. Cytokines and other mediators in inflammatory diseases lead to insulin resistance, diabetes and hyperlipidemia. Accumulating evidence in the field of immunometabolism suggests that the cause-effect relationship between arthritis and metabolic abnormalities might be bidirectional. Indeed, the immune response can be modulated by various factors such as environmental agents, bacterial products and hormones. Insulin is produced by pancreatic cells and regulates glucose, fat metabolism and cell growth. The action of insulin is mediated through the insulin receptor (IR), localized on the cellular membrane of hepatocytes, myocytes and adipocytes but also on the surface of T cells, macrophages, and dendritic cells. In murine models, the absence of IR in T-cells coincided with reduced cytokine production, proliferation, and migration. In macrophages, defective insulin signaling resulted in enhanced glycolysis affecting the responses to pathogens. In this review, we focalize on the bidirectional cause-effect relationship between impaired insulin signaling and arthritis analyzing how insulin signaling may be involved in the aberrant immune response implicated in arthritis and how inflammatory mediators affect insulin signaling. Finally, the effect of glucose-lowering agents on arthritis was summarized.

## Introduction

Insulin, the main actor of glucose homeostasis, exerts its action through the transmembrane insulin receptor (IR), expressed on target cells such as hepatocytes, adipocytes, synoviocytes, or muscle cells (1). However, IR can also be found on the membrane of T cells, macrophages or dendritic cells, and an immunoregulatory function of insulin has been suggested. Indeed, glucose is necessary for immune cells to produce energy and to maintain normal activity (2). For this reason, insulin plays a pivotal role in maintaining physiological immune response. In diabetic patients, administration of insulin may decrease levels of C-reactive protein (CRP), reduce the ability of neutrophils to generate reactive oxygen species (ROS) and suppress transcription of different Toll-like receptors (TLRs) on circulating mononuclear cells (3, 4). The interplay between inflammation, immunity and metabolism has been outlined (5) and, in this context, insulin signaling, similarly to what is observed in type 2 diabetes (T2D) or in metabolic syndrome, may be involved in the dysregulation of immune response in inflammatory diseases. Epidemiological and laboratory studies reported a possible correlation between insulin resistance and osteoarthritis (OA), rheumatoid arthritis (RA), spondyloarthritides or systemic lupus erythematosus (6–13).

In this review, we summarize the available literature about the bidirectional cause-effect relationship between impaired insulin signaling in inflammatory and degenerative arthritis, analyzing how insulin signaling may contribute to the aberrant immune response found in arthritis and how inflammatory mediators impair insulin signaling. Finally, the effect of glucose-lowering agents on arthritis was reviewed.

## Physiology of Insulin Receptor and Insulin Signaling Cascade

**Figure 1:**
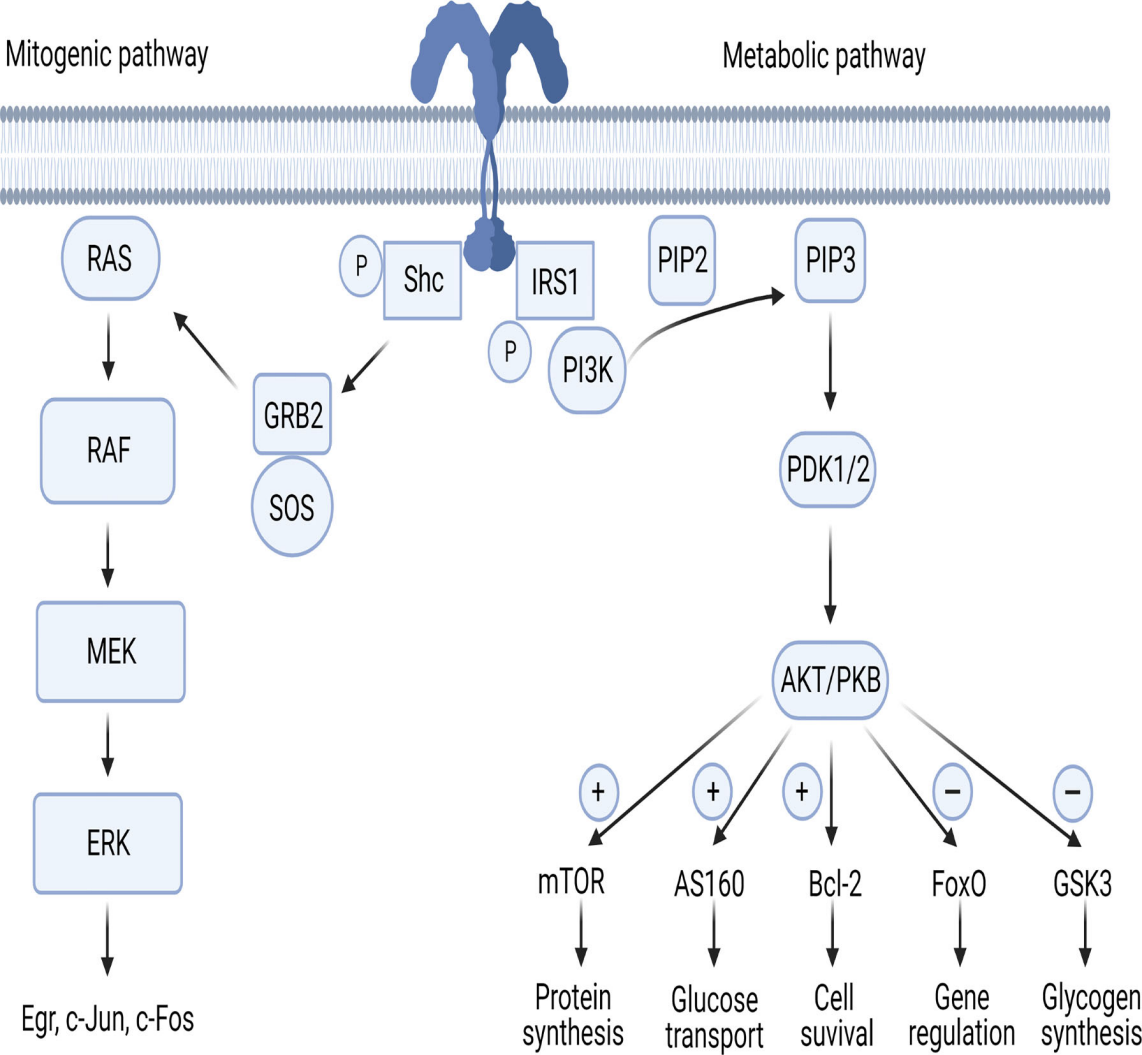
The mitogenic and metabolic pathways of insulin signaling. AKT/PKB, protein-kinase B; AS160, AKT substrate of 160kDa; Bcl-2, B-cell lymphoma 2; ERK, extracellular signal-regulated kinases; FoxO, forkhead box-containing protein O subfamily; GRB2, growth factor receptor-bound protein 2; GSK3, glycogen synthase kinase 3; IRS, insulin receptor substrate; MEK, MAPK/ERK Kinase; mTOR, mammalian target of rapamycin; PDK1/PDK2, phosphoinositide-dependent protein kinase; PI3K, phosphoinositide-3 kinase; PIP_2_, phosphatidylinositol (4,5)-bisphosphate; PIP_3_, phosphatidylinositol (3,4,5)-trisphosphate; RAF, rapidly accelerated fibrosarcoma; RAS, Rouss avian sarcoma; Shc, Src-homology collagen; SOS, son of sevenless. **Figure 1** has been created using BioRender (www.biorender.com).

The IR is a dimer located on cellular membrane and composed of four domains linked by disulfide bonds: two extracellular (α) and two intracellular (β). The α domains host the binding site of insulin, while the β domains have tyrosine kinase activity (14–17). Once circulating insulin binds the α subunits, IR undergoes autophosphorylation, with consequent phosphorylation of intracellular substrates. In the first step, IR substrates 1 and 2 (IRS-1 and IRS-2) and the docking protein Src-homology collagen (Shc) are phosphorylated, leading to the activation of two main pathways, metabolic and mitogenic (**Figure 1**). The former involves phosphoinositide3-kinase (PI3K), while the latter is mediated by mitogen activated protein kinase (MAPK) (17).

### The Metabolic Pathway

In the first step of the metabolic pathway, the PI3K regulatory subunit p85 or p55 binds to IRS-1 and IRS-2 activating the PI3K cascade. Next, the p110 catalytic subunit activation results in phosphatidylinositol-3,4,5-triphosphate (PIP3) generation leading phosphoinositide-dependent protein kinase (PDK) 1 and 2 to activate the three isoforms of AKT/PKB (**Figure 1**). PDKs become then activated after binding to PIP3 in the cell membrane (18, 19). AKT/PKB regulates five main substrates: 1- activates mammalian target of rapamycin (mTOR), responsible of protein synthesis; 2- inhibits glycogen synthase kinase 3 (GSK3), responsible of glycogen synthesis; 3- inhibits forkhead box-containing protein O subfamily (FoxO), involved in the regulation of gluconeogenic and adipogenic genes; 4- increases the AKT substrate of 160kDa (AS160), responsible of glucose transport (20, 21); 5- upregulates Bcl-2 expression, involved in cell survival (22).

### The Mitogenic Pathway

In the first step of the mitogenic pathway, growth factor receptor-bound protein 2 (GRB2) is activated by phosphorylated Shc protein (**Figure 1**). GRB2 acts as a bond that links IRS-1 to son-of-sevenless (SOS), which is a guanine nucleotide exchange factor. GRB2/SOS promotes exchange of GDP with GTP on Rouss avian sarcoma (Ras), thus activating it (23). Activated Ras recruits Raf serine/threonine protein kinase and then the MAPK pathway transcription factors MEK, ERK and p90 inducing the activation of the Egr genes c-Jun and c-Fos (24, 25).

## Insulin, Immune Cells and Arthritis

Immune cells need glucose to produce energy (14) and, similar to adipose, muscle and liver cells, also immune cells express IR on their surface (26, 27). Through IR, insulin acts as a glucose-regulating hormone and behaves as a growth-like factor and cytokine regulator (28–30), exerting its immunomodulatory effects (2, 31).

**Figure 2:**
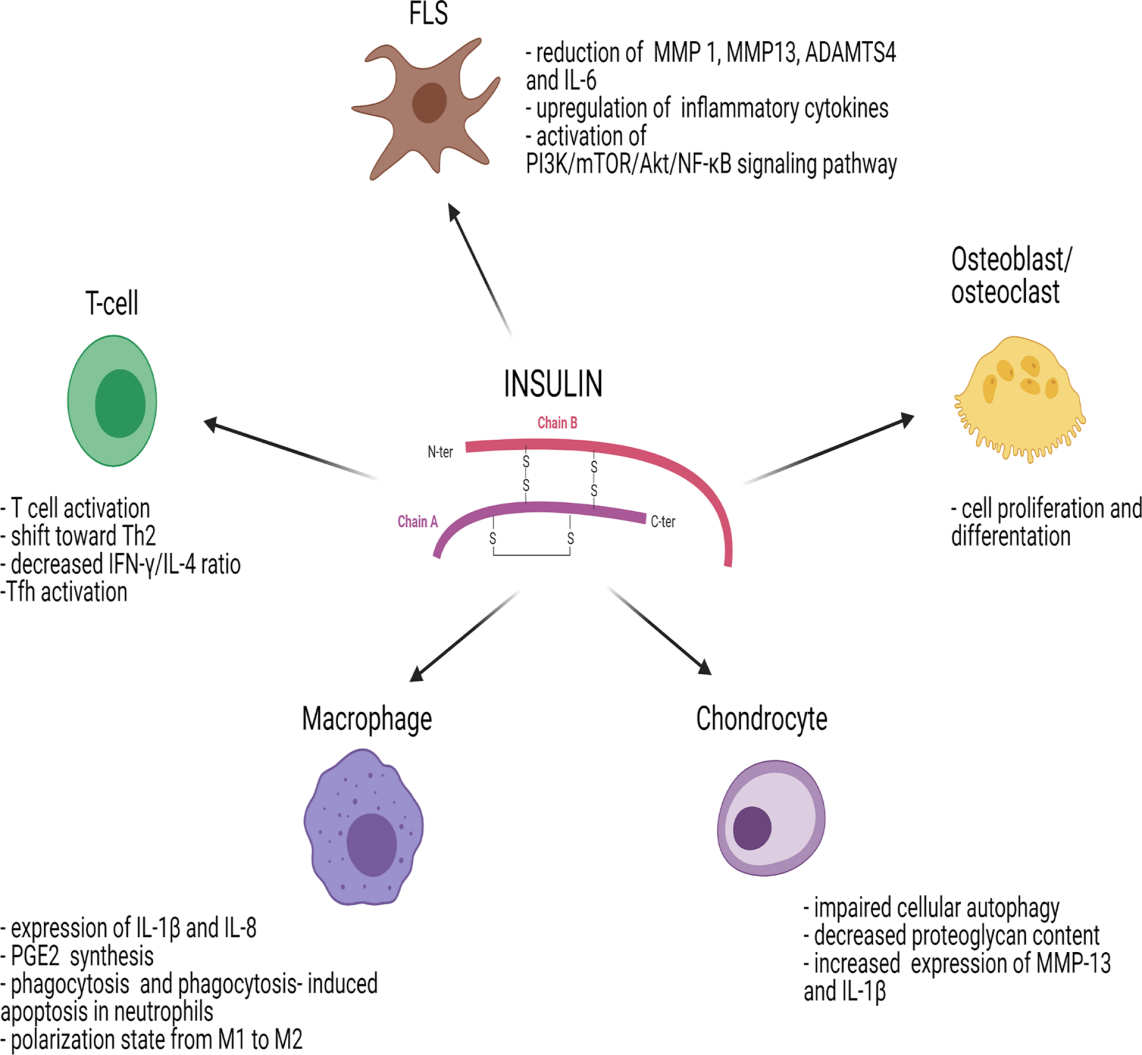
Effects of insulin on immune cells and joint cells. ADAMTS4, metalloproteinase with Thrombospondin Type 1 Motif 4; AKT/PKB, protein-kinase B; FLS, fibroblast-like synoviocyte; IL, interleukin; IFN-γ, interferon gamma; MMP, matrix metalloproteinase; mTOR, mammalian target of rapamycin; NF-κB, nuclear factor kappa-light-chain-enhancer of activated B cells; PGE2, prostaglandin E2; PI3K, phosphoinositide-3-kinase; Tfh, follicular helper T cells; Th2, T helper 2. **Figure 2** has been created using BioRender (www.biorender.com).

Insulin modulates the immune response either indirectly through the glucose-lowering effect or directly by acting on immune cells and influencing their proliferative responses and signal transduction (32) (**Figure 2**). Regarding the first point, hyperglycemia has negative effects on the immune system since it induces cell stress and leads to the generation of advanced glycation end products (AGEs) and ROS, which stimulate release of various pro-inflammatory mediators. It can therefore be hypothesized that insulin, through its glucose-lowering role, reduces “glucose toxicity” and cell stress, exerting an anti-inflammatory effect (33).

In addition to these actions on glucose metabolism, insulin exerts anti-inflammatory effects *via* the stimulation of various intercellular pathways. Activation of PI3K/Akt pathway reduces the transcriptional activity of FoxO proteins that in turn suppress TLR4 signaling in response to lipopolysaccharides (LPS) in leukocytes (34) leading to a downregulation of the immune system. Furthermore, insulin antagonizes the pro-inflammatory transcriptional activity of nuclear factor kappa-light-chain-enhancer of activated B cells (NF-κB) and activates the mTOR complex (mTORC), enhancing p62 phosphorylation and then p62-mediated degradation of the Kelch-like ECH-associated protein 1 (Keap1), thus allowing antagonism of pro-inflammatory processes mediated by nuclear factor erythroid 2-related factor 2 (Nrf2). Lastly, insulin inhibits the transcription of various TLRs on circulating mononuclear cells, including TLR1, 2, 4, 7 and 9 (35), causing a decreased immune response.

On the other hand, several investigations suggested a role of insulin as pro-inflammatory hormone. Studies in healthy, nondiabetic subjects explored the effects of insulin on polymorphonuclear (PMN) leukocytes functions. In vivo experiments with hyperinsulinemic clamp demonstrated that insulin stimulated PMN chemotaxis ability and phagocytosis but it didn’t affect ROS production or density of surface receptors such as IR, CD11b, CD15, CD62L and CD89 (36). Similar experiments in monocytes demonstrated a suppressive action of insulin on formyl-methionyl-leucyl-phenylalanine-induced ROS production. Furthermore, again in monocytes, insulin inhibits in a dose‐dependent manner the upregulation of tissue factor procoagulant activity. The inhibition is caused by a mechanism that interferes with the regulation of cyclic AMP and intracellular calcium, independently of the PI3K–PKB pathway (37).

RA is an autoimmune inflammatory joint disease often characterized by the presence of rheumatoid factor (RF) and anti-citrullinated protein antibodies (ACPAs). Epidemiological studies suggested a direct relationship between diabetes and rheumatoid arthritis especially in female patient (38).

Treg cells mediate tolerance to self-antigens, whereas Th17 cells are involved in the pro-inflammatory reaction to pathogens. In RA and in other autoimmune diseases, the Treg-Th17 equilibrium is altered. Glycolytic pathways and increased glucose consumption lead to a metabolic switch from low-energy to highly active state in RA (39). Insulin and insulin‐like growth factors (IGFs) are similar polypeptide hormones. Insulin regulates the use of carbohydrates, while IGF‐1 is involved in cell growth, differentiation, and survival. Insulin and IGFs use signaling pathways involving PI3K and Akt or Ras and MAPK, which are also involved in other cellular stimuli (40). RA is characterized by an alteration of IGF-1 axis and its receptor (IGF-1R), which is expressed on chondrocytes, synovial fibroblasts and leukocytes (41–43). In RA patients there is upregulation of the IGF-1R expression on CD4+ T cells compared to healthy controls and in RA patients the IGF-1 levels are lower (44).

Moreover, the proinflammatory adipokine resistin, involved in obesity and diabetes, has a relevant role in the pathogenesis of RA and in the induction of the inflammatory response (45). When resistin is suppressed, the result is reduced expression of IGF‐1R and decreased phosphorylation of Akt (42). Resistin modulates Akt‐dependent processes and IGF‐1R expression in human synovial tissue interfering with IR/IGF‐1R signaling.

Similar to insulin, IGF-1R signaling plays a role in inflammation mediated by T cells in arthritis. IGF-1R has an inhibitory effect on the level of IR substrates, reducing IL6-dependent formation of Th17 cells. The effects of insulin on different immune cells and joint cells are summarized in **Figure 2**.

### Insulin Signaling in T Cells

While IR can be detected on the surface of B cells, monocytes and resting neutrophils, it is not expressed on resting T cells (36, 46). However, IR is significantly upregulated on activated T cells (47, 48) and it is essential to meet the large glucose demand that T cells need to acquire full effector functions. Insulin signaling in T cells promotes T cell activation by increasing protein synthesis, glucose uptake and amino acid transport (49). Viardot et al. demonstrated *in vitro* that insulin induced a shift toward T helper type 2 (Th2) response, reducing the T helper type 1 (Th1) to Th2 ratio (**Figure 2**). This resulted in a change of cytokine secretion with decreased interferon-gamma to IL-4 ratio with enhanced phosphorylation of extracellular signal-regulated kinase (ERK) (48), one of the four MAPK signaling pathways.

Experiments on IR knockout mice demonstrated that there was an impairment of polyclonal activation of CD4+ T cells and of cytokine production, migration and proliferation (50). Similar results were observed also in CD8+ T cells showing impaired cytotoxicity in response to alloantigens. Studies on obese patients have outlined that insulin resistance and related disorders are characterized by a cytokine imbalance, with high levels of TNFα, IL-6, IL-1β, CRP, and NF-κB (51). In this regard, Tao et al. found an imbalance between Th17 and regulatory T cells (Treg) in insulin resistance state (52). Th17 and Treg represent two CD4+ T cell subsets that share some developmental elements but express different phenotypes with opposite actions. The former have pro-inflammatory activity and the latter show anti-inflammatory activity (53). An altered balance between Treg and Th17 cells is involved in RA and other immune-mediated conditions (54).

Stimulation of the T cell receptor (TCR) complex and ligation of the co-receptor CD28 by co-stimulatory molecules activate quiescent T cells (55). Engagement of the TCR stimulates intracellular signaling through the ERK/MAPK pathways, while the PI3K-Akt-mTOR axis is activated by CD28 signaling (56, 57). PI3K-Akt signaling activates glycolysis and increases glucose transporter 1 (Glut1) expression and thus glucose uptake. Overexpression of Glut1 enhances T follicular helper cells (Tfh) differentiation, a T cell subset involved in B cell regulation (58), potentially favoring autoimmunity in both type 1 diabetes and RA (59). Downstream, PI3K-Akt activates mTOR, which promotes the differentiation of Th1, Th17 and Tfh cells (60). Furthermore, mTOR can inhibit the formation of long-lived Tregs favoring effector Tregs (61). Treg knockout for mTOR reduced their frequency, leading to spontaneous effector T cell activation and inflammation (62).

AMP-activated protein kinase (AMPK) can inhibit cellular growth *via* suppression of the mTORC1 pathway (63). AMPK activation and interruption of mTOR signaling mitigated the inflammation in experimental arthritis. AMPK-dependent control of fatty acid metabolism may also impact cell fate decisions in CD4+ T cells, particularly the balance between Th17 and Treg lineages (64).

In addition, growth factors such as insulin, IGF-1 or IL-2 can stimulate PI3K-Akt-mTOR signaling. The roles played by insulin and IGFs are different, but they share the PI3K-AKTmTOR and RAS-RAF-MEK-ERK signaling pathways. Signaling through IGF-1 receptor (IGF1R) activates the Akt-mTOR pathway, increases aerobic glycolysis and ultimately favors Th17 cell differentiation over Treg cells. In experimental models, the inhibition of IGF-1R signaling may improve arthritis by decreasing IL6 production and modifying the balance between Th17 and Treg generation dependent on IL6 (44).

### Insulin Signaling in Synoviocytes

Insulin signaling plays an important role in synoviocytes, which express a large number of IRs. In synoviocytes, insulin promotes the inflammatory phenotype of fibroblast-like synoviocytes (FLSs), increases cell viability, promotes production of inflammatory cytokines and chemokines and facilitates chemotaxis of macrophages, leading to synovial membrane inflammation (**Figure 2**). In synovial tissue isolated from patients with OA, a condition with persistent low-grade inflammation, and T2D, there was phosphorylation of Akt and reduced autophosphorylation of the IR induced by insulin. In OA patients with T2D, insulin resistance may develop not only in insulin-sensitive tissues such as muscle, liver or fat, but also in the synovial membrane. Furthermore, Hamada et al. demonstrated that insulin markedly reduced TNFα-stimulated production of matrix metalloproteinase (MMP) 1, MMP13, ADAMTS4, BMP2 and IL-6 in nondiabetic human FLSs without reducing TNFα itself, implicating a pivotal role of insulin in the inhibition of synovial inflammation (6, 65).

Qiao et al. showed that, in FLSs, insulin activates the PI3K/mTOR/Akt/NF-ĸB signaling pathway and inhibits autophagy. Insulin can also upregulate inflammatory cytokine receptors whereas PI3K/mTOR/Akt/NF-ĸB signaling inhibitors can reverse this process in FLSs (66).

### Insulin Signaling in Osteoblasts and Osteoclasts

Previous studies supported the role of insulin signaling in the biology and pathology of the joint (67), mainly through its capability to control bone architecture acting on osteoblasts and osteoclasts (68–73) (**Figure 2**). In vitro experiments demonstrated that insulin upregulated IR expression. Moreover, through MAPK and PI3K pathway, insulin stimulated cell proliferation and differentiation by increasing alkaline phosphatase activity, secretion of type I collagen and expression of osteocalcin in MG-63 cells (69). In IR knockout mice, increased expression of osteoprotegerin in osteoblasts and inhibited osteoclastogenesis and osteoclastic activity were observed (74, 75). The activation of mTORC1 by IGF-1, which is released in the bone resorption phase, stimulates osteoblast differentiation of mouse bone marrow stromal cells (BMSC) (76). mTORC1 is in fact required for the transition of pre-osteoblasts to mature osteoblasts (77).

However, insulin acts also on osteoclasts. Through the ERK1/2 pathway, insulin induces the upregulation of receptor activator of nuclear factor-κB (RANK) contributing to the enhancement of osteoclast differentiation by RANKL (78). The effects of mTORC1 on osteoclasts have not been completely elucidated. In osteoclast precursors, the deletion of *raptor* leading to inactivation of mTORC1, or the activation of mTORC1 by deletion of tuberous sclerosis complex 1 (Tsc1), could respectively increase or reduce osteoclastogenesis. Mechanistically, this was due to mTORC1 inhibition of NF-kB and nuclear factor of activated T Cells 1 (NFATc1), both critical transcription factors of osteoclastogenesis (79). Another study shows how RANK ligand (RANKL)-dependent osteoclastogenesis is impaired in Tsc1-deficient bone marrow macrophages, where TSC1 is a negative regulator of mTORC1 (80).

Dai et al. suggested that in bone marrow macrophages, inhibition of mTORC1 by treatment with rapamycin or by genetic deletion, suppressed *in vitro* osteoclast differentiation rescued by upregulation of mTOR downstream target S6K159 (81). Collectively, these studies outline how the insulin/mTOR pathway plays a role in bone biology, however further investigation is needed to properly dissect its anabolic and catabolic role.

### Insulin Signaling in Chondrocytes

Insulin resistance and hyperinsulinemia were shown to be involved in the pathogenesis of OA and metabolic syndrome (82, 83) (**Figure 2**). In human chondrocytes, insulin increases the mTOR signaling pathway and Akt phosphorylation in a dose-dependent manner, leading to impaired cellular autophagy, an important mechanism regulating the removal and degradation of damaged intracellular products (84). Treatment with rapamycin, an mTOR inhibitor, reversed the effects of insulin on autophagy activity and beneficial effects on cartilage integrity were observed (85). Furthermore, insulin reduced the content of proteoglycans and upregulated MMP-13 and IL-1β, which have a significant role in chondrocytes and in cartilage degradation (84, 86).

### Insulin Signaling in Macrophages

During insulin resistance state, Akt signaling is impaired leading to hyperactivation of mTORC1 and increased glycolysis. In macrophages, increased glycolysis affects responses to pathogens and danger signals (87). Insulin significantly enhances the LPS-dependent expression of IL-1β and IL-8 and the induction of enzymes involved in the prostaglandin E2 (PGE2) synthesis by macrophages (88) (**Figure 2**). In vivo and *in vitro* studies suggested that insulin re-established phagocytosis and fostered phagocytosis-induced apoptosis in neutrophils. Furthermore, insulin treatment induced macrophages to change their polarization state from M1 to M2 (87).

### Insulin Resistance and Arthritis

Prevalence of insulin resistance is increased in RA patients (89–92) and it is correlated with disease activity and disease-specific factors such as chronic systemic inflammation and use of glucocorticoids which may cause dysfunction of pancreatic β cells (90, 93, 94). Indeed, it has been hypothesized that the glucose intolerance observed in RA is contributed by the inefficacy of β cells to compensate for insulin resistance (93) and that, in β cells of non-diabetic RA patients not receiving glucocorticoids, there is an impairment of proinsulin to insulin processing possibly explained by the sustained pro-inflammatory state (95).

Furthermore, in OA, it has been suggested that pathophysiological mechanisms similar to those observed in T2D are present and insulin resistance-related traits might have a role in the development of the disease (96).

## Glucose-Lowering Agents and Arthritis

Increasing evidence suggests that glucose-lowering agents exert a number of anti-inflammatory activities (97). The anti-arthritis effects of metformin, thiazolidinediones (TZDs), dipeptidyl peptidase-4 (DPP-4) inhibitors and glucagon-like peptide-1 (GLP-1) receptor agonists were investigated in several studies and are summarized in **Table 1**. Currently, no data exist about the sodium-glucose cotransporter type 2 (SGLT2) inhibitors.

**Table 1:** Effects of glucose-lowering agents on arthritis.

**Metformin**	
• antiproliferative, antifibrotic, and antioxidant potential	
• ↓ Th17 cells and ↓ proinflammatory cytokines	
• inhibition of mitochondrial respiratory chain complex I and ↓ ROS	
• ↓ STAT3 phosphorylation *via* AMPK/mTOR pathway and ↓ Th17 differentiation	
• phosphorylation of AMPK	
• in synovial fibroblasts ↑ glycolytic activity and ↓ IL 6, IL 8 and monocyte chemotactic protein 1	
• in osteoclasts ↓ osteoclastogenesis by the AMPK-mediated inhibition of mTOR	
• in macrophages ↓ of TNFα, IL-6, and MCP-1; ↑ release of IL-10	
• ↓ inflammatory cytokines by suppressing NF-κB pathway	
**Thiazolidinediones**	
• anti-inflammatory activity, immuno-modulation, antioxidant effect	
• in macrophages ↓ proliferation	
• in T-cells ↑ immunosuppressive effects	
• ↓ production of IL-17; ↓ mRNA expression levels of inflammatory mediators; ↓ levels of MMPs	
• in synoviocytes/synovial fibroblast ↓ growth and ↓ IL 1β induced PGE2 synthesis	
• maintained expression of aggrecan and type II collagen	
• ↓ inflammatory cell infiltration, ↓ pannus formation, ↓ cartilage/bone damage	
• ↓ TNFα, IL-1β, MCP-1 and RANKL mRNA, ↓ osteoclasts differentiation	
• in chondrocytes ↓ NO synthase expression, ↓ IL-1β and MMP-13	
• in chondrocytes/synovial fibroblasts ↓ COX-2 expression and PGE2 production	
• ↓ NF-κB pathway	
**Dipeptidyl peptidase-4 inhibitors**	
• ↓ proliferation of T cells	
• ↓ anti-CCP, RANKL, TNFα and IL-6 by ↓TLR/NF-κB pathway	
• action on cytokine secretion, T cell‐dependent antibody production and immunoglobulin isotype switching of B cells	
• in chondrocytes ↓ degradation of type II collagen by MMP-1, MMP-3, and MMP-13	
• ↓ oxidative stress	
• ↓ ADAMTS-4 and ADAMTS-5 leading to ↓ degradation of aggrecan	
• ↓ p38 MAPK signaling pathway and ↓ NF-κB	
**Glucagon-like peptide 1 analogues**	
• in FLSs ↓ TNFα, IL-6, IL-8, IL‐1β, MMP‐3, MMP‐13, HMGB-1, MCP‐1, p38/MAPK and NF‐κB pathways	
• in FLSs improved oxidative stress and prevented cell death	
• in chondrocytes ↑ anti-apoptotic marker Bcl-2 and ↓ apoptotic proteins active caspase 3 and Bax	
• in chondrocytes ↑ deterioration of type II collagen and aggrecan	

### Metformin

Metformin represents the first line treatment of T2D and insulin resistance states. Besides its anti-hyperglycemic effects, metformin has antiproliferative, antifibrotic, and antioxidant potential (98, 99) (**Table 1**). The main mediator of the anti-inflammatory properties of metformin is AMPK activation, which controls inflammation and immunity through a variety of mechanisms (100). The anti-inflammatory effects of metformin are also independent from AMPK. Indeed, metformin is a potent inhibitor of mitochondrial respiratory chain complex I (NADH: ubiquinone oxidoreductase) (101, 102) which is implicated in the production of ROS (103). Metformin was found to decrease IL-1β and to boost IL-10 as well as to inhibit ROS production in LPS-activated murine macrophages in an AMPK independent manner (103).

In a mouse model of autoimmune arthritis, metformin downregulated Th17 cells decreasing proinflammatory cytokines and inhibiting the differentiation of Th17 differentiation through inhibition of STAT3 phosphorylation (104).

The phosphorylation of AMPK induced by metformin may partially improve synovial inflammation in RA. Under physiologic conditions, phosphorylation of AMPK reconstitutes cell stores of ATP, generating new ATP and inhibiting the inflammatory pathways, which are energy-expensive (105, 106). In RA synovial fibroblasts, metformin increases glycolytic activity and decreases oxidative phosphorylation and generation of IL‐6, IL‐8 and monocyte chemotactic protein 1. Metformin can also suppress the differentiation of osteoclasts and the AMPK-mediated inhibition of mTOR negatively regulates osteoclastogenesis (107). In vitro, metformin can act on macrophages to inhibit the release of TNFα, IL-6, and MCP-1 while enhancing IL-10. In vivo, metformin can reduce inflammatory cytokine production leading to clinical improvement of arthritis. These effects were exerted by correcting the impaired autophagic flux and selectively degrading IκB kinase causing suppression of NF-κB-mediated signaling (108).

### Thiazolidinediones

TZDs act as insulin sensitizing agents in liver, fat and skeletal muscle cells, through the activation of the nuclear peroxisome proliferator-activated receptor γ (PPARγ). When PPAR-γ is activated, insulin-responsive genes controlling glucose and lipid metabolism are transcribed. Similar to metformin, also TZDs have pleiotropic properties including anti-inflammatory activity, immuno-modulation and antioxidant effects (109–114) (**Table 1**). From a molecular point of view, the anti-inflammatory action is exerted through the inhibition of NF-κB signal pathway (115). PPARγ acts as an E3 ubiquitin ligase, physically interacting with NF-κB p65 subunit to induce its ubiquitination and degradation thus limiting pro-inflammatory cytokine production (116).

Interestingly, PPAR-γ ligands seem to have an immunomodulatory role on monocytes and macrophages. Pioglitazone suppressed macrophage proliferation without inducing apoptosis (117, 118). Furthermore, PPARγ ligation induces T-cell immunosuppressive effects (118, 119).

The role of pioglitazone has been investigated in models of IL-17-induced human intervertebral disc degeneration. Its administration reduced the levels of inflammatory cytokines such as IL-17, and downregulated mRNA expression of inflammatory mediators. Furthermore, pioglitazone suppressed MMPs and preserved the expression of the extracellular matrix molecules aggrecan and type II collagen (120).

Anti-inflammatory effects of TZDs were demonstrated also in models of RA. In vitro, the growth of RA synoviocytes is inhibited by PPAR‐γ ligands which also downregulate, in RA synovial fibroblasts, the synthesis of PGE2 mediated by IL‐1β (121). Indeed, available evidence suggests that synthetic PPARγ agonists such as rosiglitazone and troglitazone, but also the natural PPARγ ligand 15-deoxy-Delta (12,14)-prostaglandin J2, can improve arthritis in murine models (122, 123).

In another set of experiments, it has been suggested that using methotrexate in combination with pioglitazone may have a synergistic effect in RA combining inhibition of inflammatory cytokines TNFα, IL-1β and prevention of the activation of ROS (124, 125).

Tomita et al. explored the effects of THR0921, a PPAR‐γ ligand, in mice models of collagen-induced arthritis. Compared with normal mice, those treated with THR0921 had milder synovial hyperplasia, with no pannus formation, lower degree of inflammatory cell infiltration and modest damage to cartilage and bone (126). Furthermore, where joint damage was observed, the expression levels of TNFα, IL-1β, MCP-1 and RANKL mRNA were reduced. Koufany et al. explored the role of oral treatment with pioglitazone and rosiglitazone in murine models with adjuvant-induced arthritis, observing decreased expression of IL-1β and TNFα in inflamed synovial tissue (127). Treatment with PPAR-γ ligands inhibits nitric oxide synthase expression induced by IL-1β in OA patients’ chondrocytes. PPAR-γ ligands reduced IL-1β and MMP-13 production in a dose-dependent manner. The inhibitory effect of PPAR-γ activation was also demonstrated on the generation of nitric oxide and MMP-13 induced by TNFα and IL-17 (128). Finally, in human chondrocytes and synovial fibroblasts, the activation of PPAR-γ decreases IL-1β induced COX-2 expression and production of PGE2 (129). Since high levels of COX-2-induced prostaglandins are associated with the generation of free radicals and lipid peroxide (130), pioglitazone exerts antioxidative and anti-inflammatory effects also through modulation of COX-2. Moreover, PPAR-γ agonists can inhibit the NF-κB pathway, having an anti-arthritic role (109).

### Dipeptidyl Peptidase-4 Inhibitors

DPP‐4 inhibitors have been widely used in T2D. They act by reducing the degradation of the incretin hormones inhibiting the DPP‐4 enzymes (**Table 1**). Incretins lead to improved glycemic control by delaying satiety, favoring the release of insulin, inhibiting the production of glucagon and preserving β‐cell mass. DPP‐4 can modify the functioning of immune system and also disease pathogenesis interfering with mechanisms of T cells development and migration but also with cytokine secretion, T cell‐dependent antibody production and immunoglobulin isotype switching (131, 132). In large, prospective, population‐based cohort studies, the risk of developing an autoimmune disease was lower in patients receiving antidiabetic therapy with DPP-4 inhibitors compared to T2D patients not treated with DPP-4 inhibitors (133, 134). In a series of pioneering studies conducted *in vivo*, Tanaka et al. demonstrated that DPP-4 inhibitors suppressed inflammation in two murine models of collagen-induced and alkyldiamine-induced arthritis, with pathological characteristics similar to RA. In particular, the inhibition of DPP-4 reduced mitogen-induced and antigen-induced proliferation of T cells (135, 136).

Ibrahim et al. (137) evaluated the effects of a combination therapy with sitagliptin and tofacitinib on JAK/STAT and TLR-4/NF-κB pathways in murine models of adjuvant-induced arthritis. The separate administration of both tofacitinib and sitagliptin resulted in a reduction of anti-CCP, RANKL, TNFα and IL-6 compared to untreated mice, but the combination of both drugs produced a more significant decrease of the serological markers compared to each therapy alone (137).

Hu et al. demonstrated the anti-inflammatory effects of saxagliptin on articular chondrocytes exposed to AGEs. Saxagliptin reduced the expression of mRNA of enzymes such as MMP-1, MMP-3 and MMP-13, which are involved in type II collagen degradation. Furthermore, it significantly inhibited expression of ADAMTS-4 and 5, resulting in less aggrecan degradation. Oxidative stress was reduced by the inhibition of DPP-4 by inhibiting ROS generation and increasing levels of glutathione. Exposure to AGEs activated the p38 MAPK signaling pathway and increased the degradation of IκBα, upregulating NF-κB. In OA, saxagliptin was able to affect this pro-inflammatory process (138).

### Glucagon-Like Peptide 1 Analogues

GLP-1 is an incretin mimetic hormone exerting different effects on glucose metabolism (**Table 1**). It can increase the secretion of insulin induced by glucose, delay gastric emptying and stimulate satiety. Moreover, GLP-1 plays a diuretic role and modulates the proliferation of β-cells (139). Growing evidence suggests that gut hormones act as key signals in regulating the interplay between the metabolic axis and the immune system and may also be involved in the response to immunomodulatory therapy for RA (140). In RA patients, the incretin-insulin axis and the incretin effect are impaired (64). The first study exploring the effects of GLP-1 analogues on the pathological characteristics of RA in human FLSs was conducted using lixisenatide. Lixisenatide downregulated TNFα, IL-6, IL-8 and MMPs, inhibiting the inflammatory response through blockage of cellular signaling pathways such as c-Jun N-terminal kinase (JNK), activator protein 1 (AP-1) and NF-κB. Moreover, treatment with lixisenatide caused a reduction in oxidative stress and prevented cell death in FLSs (141).

Chen et al. explored the role of GLP-1 receptor (GLP-1R) in OA demonstrating that liraglutide could protect chondrocyte apoptosis and extracellular membrane degradation by regulating endoplasmic reticulum stress. Liraglutide upregulated the anti-apoptotic marker Bcl-2 and diminished the expression of apoptotic proteins active caspase 3 and Bax (142). The activation of GLP-1R/PI3K/Akt signaling by GLP-1 analogues is involved in various metabolic processes. The inhibition of PI3K/Akt signaling impaired the protective effects of GLP-1R increasing apoptotic activity and endoplasmic reticulum stress. The activation of GLP-1R inhibited the NF-κB pathway thus decreasing the release of inflammatory mediators. The same results were obtained also in a model of knee OA (142).

Tao et al. studied the role in RA pathogenesis of GLP-1R on human FLSs using the selective GLP-1 agonist exenatide. FLSs were exposed to TNFα in the presence or absence of exenatide. Exenatide treatment significantly reduced expression of IL‐1β, IL‐6, MMP‐3, MMP‐13 and MCP‐1. Moreover, by preventing IκBα degradation, the treatment inhibited activation of the p38/MAPK and NF‐κB pathways (143).

Similar results were observed in RA human FLSs incubated with TNF in presence of dulaglutide. Dulaglutide treatment significantly downregulated proinflammatory mediators such as IL-1β, IL-6, MCP-1, HMGB-1, MMP-3 and MMP-13. The effects of dulaglutide were mediated by the inactivation of JNK and increased phosphorylation of IκBα, causing a reduction of NF-κB (144).

The inhibitory effect of dulaglutide on OA-related cytokines and chemokines was demonstrated also in chondrocytes treated with AGEs. In chondrocytes, the AGEs-mediated deterioration of articular extracellular matrix components, such as type II collagen and aggrecan, was reduced by treatment with dulaglutide through inhibition of MMP-3 and MMP-13 (145).

## Effects of Disease-Modifying Antirheumatic Drugs on Glucose Metabolism

Compared with the general population, insulin resistance is more prevalent in patients affected by RA (7, 90, 92, 146, 147). Insulin resistance is influenced by both inflammation-related and metabolic factors (95, 147–149) but, in RA patients, it can be modified by the use of anti-rheumatic drugs. The cytokines involved in the pathogenesis of RA, in particular TNFα, IL-1 and IL-6, also promote the development of insulin resistance. The topic has been extensively reviewed elsewhere (7, 9, 150) but, briefly, by acting through IRS-1, TNF-α reduces IR tyrosine kinase activity and induces serine phosphorylation leading to inhibition of IR in skeletal muscle cells and adipocytes (151, 152). Anti-TNFα agents improve insulin sensitivity and decrease insulin resistance in RA patients, also reducing the risk of developing T2D (153–157). Moreover, similar effects on the decrease of insulin resistance were shown in RA patients treated with IL-6 antagonists (158–160), anti-IL-1 agents (161, 162) or T-cell costimulation blockade (163). In summary, the introduction of disease-modifying anti-rheumatic drugs can control inflammation and exert beneficial effects on insulin resistance and insulin sensitivity in RA patients, potentially reducing the risk of developing T2D in non-diabetic individuals or aiding in the achievement of better glucose control in diabetics.

## Conclusions

In summary, through its integrated signaling network, insulin regulates intracellular and intercellular pathways in immune cells, in cartilage and in synovial tissue, behaving as a crucial modulator of the inflammatory response observed in arthritis. Finally, robust *in vitro* and *in vivo* evidence outlines the effects of glucose-lowering therapies in arthritis. Metformin, TZDs, DPP-4 inhibitors and GLP-1 analogues may exert an immunomodulatory action downregulating the expression of proinflammatory cytokines and chemokines, thus reducing synovial inflammation and potentially leading to improvement of arthritis.

## Author Contributions

CT, CB, RG, RM, and FU contributed to conception of the review. CT, JC, VP, PR, and NV performed the literature search and wrote sections of the manuscript. All authors contributed to manuscript revision, read, and approved the submitted version.

## Conflict of Interest

The authors declare that the research was conducted in the absence of any commercial or financial relationships that could be construed as a potential conflict of interest.

## References

[1] Haeusler RA McGraw TE Accili D. Biochemical and Cellular Properties of Insulin Receptor Signalling. Nat Rev Mol Cell Biol (2018) 19(1):31–44. 10.1038/nrm.2017.89 28974775 PMC5894887

[2] van Niekerk G Christowitz C Conradie D Engelbrecht AM. Insulin as an Immunomodulatory Hormone. Cytokine Growth Factor Rev (2020) 52:34–44. 10.1016/j.cytogfr.2019.11.006 31831339

[3] Shoelson SE Lee J Goldfine AB. Inflammation and Insulin Resistance. J Clin Invest (2006) 116(7):1793–801. 10.1172/JCI29069 PMC1483173 16823477

[4] Lin Y Berg AH Iyengar P Lam TK Giacca A Combs TP. The Hyperglycemia-Induced Inflammatory Response in Adipocytes: The Role of Reactive Oxygen Species. J Biol Chem (2005) 280(6):4617–26. 10.1074/jbc.M411863200 15536073

[5] Pucino V Certo M Varricchi G Marone G Ursini F Rossi FW. Metabolic Checkpoints in Rheumatoid Arthritis. Front Physiol (2020) 11:347. 10.3389/fphys.2020.00347 32362840 PMC7180190

[6] Griffin TM Huffman KM. Editorial: Insulin Resistance: Releasing the Brakes on Synovial Inflammation and Osteoarthritis? Arthritis Rheumatol (2016) 68(6):1330–3. 10.1002/art.39586 PMC5371020 26749517

[7] Nicolau J Lequerré T Bacquet H Vittecoq O. Rheumatoid Arthritis, Insulin Resistance, and Diabetes. Joint Bone Spine (2017) 84(4):411–6. 10.1016/j.jbspin.2016.09.001 27777170

[8] Chen HH Yeh SY Chen HY Lin CL Sung FC Kao CH. Ankylosing Spondylitis and Other Inflammatory Spondyloarthritis Increase the Risk of Developing Type 2 Diabetes in an Asian Population. Rheumatol Int (2014) 34(2):265–70. 10.1007/s00296-013-2927-5 24362789

[9] Ursini F Russo E Ruscitti P Giacomelli R De Sarro G. The Effect of non-TNF-targeted Biologics and Small Molecules on Insulin Resistance in Inflammatory Arthritis. Autoimmun Rev (2018) 17(4):399–404. 10.1016/j.autrev.2017.11.030 29452240

[10] Ursini F D’Angelo S Russo E Arturi F D’Antona L Bruno C. Serum Complement C3 Strongly Correlates With Whole-Body Insulin Sensitivity in Rheumatoid Arthritis. Clin Exp Rheumatol (2017) 35(1):18–23. 27908300

[11] Ursini F D’Angelo S Russo E Nicolosi K Gallucci A Chiaravalloti A. Complement C3 is the Strongest Predictor of Whole-Body Insulin Sensitivity in Psoriatic Arthritis. PloS One (2016) 11(9):e0163464. 10.1371/journal.pone.0163464 27656896 PMC5033360

[12] García-Dorta A Quevedo-Abeledo JC Rua-Figueroa Í de Vera-González AM González-Delgado A Medina-Vega L. Beta Cell Function is Disrupted in Patients With Systemic Lupus Erythematosus. Rheumatol (Oxford) (2020). 10.1093/rheumatology/keaa874 33369681

[13] Sánchez-Pérez H Tejera-Segura B de Vera-González A González-Delgado A Olmos JM Hernández JL. Insulin Resistance in Systemic Lupus Erythematosus Patients: Contributing Factors and Relationship With Subclinical Atherosclerosis. Clin Exp Rheumatol (2017) 35(6):885–92. 28281456

[14] De Meyts P. The Insulin Receptor and Its Signal Transduction Network. In: Feingold KR Anawalt B Boyce A Chrousos G de Herder WW Dungan K, editors. Endotext. South Dartmouth (MA: MDText.com, Inc. Copyright © 2000-2021, MDText.com, Inc (2000). 27512793

[15] Belfiore A Malaguarnera R Vella V Lawrence MC Sciacca L Frasca F. Insulin Receptor Isoforms in Physiology and Disease: An Updated View. Endocr Rev (2017) 38(5):379–431. 10.1210/er.2017-00073 28973479 PMC5629070

[16] Hubbard SR Wei L Ellis L Hendrickson WA. Crystal Structure of the Tyrosine Kinase Domain of the Human Insulin Receptor. Nature (1994) 372(6508):746–54. 10.1038/372746a0 7997262

[17] Petersen MC Shulman GI. Mechanisms of Insulin Action and Insulin Resistance. Physiol Rev (2018) 98(4):2133–223. 10.1152/physrev.00063.2017 PMC6170977 30067154

[18] Muniyappa R Montagnani M Koh KK Quon MJ. Cardiovascular Actions of Insulin. Endocr Rev (2007) 28(5):463–91. 10.1210/er.2007-0006 17525361

[19] Liu P Cheng H Roberts TM Zhao JJ. Targeting the Phosphoinositide 3-Kinase Pathway in Cancer. Nat Rev Drug Discovery (2009) 8(8):627–44. 10.1038/nrd2926 PMC3142564 19644473

[20] Huang X Liu G Guo J Su Z. The PI3K/AKT Pathway in Obesity and Type 2 Diabetes. Int J Biol Sci (2018) 14(11):1483–96. 10.7150/ijbs.27173 PMC6158718 30263000

[21] Taniguchi CM Emanuelli B Kahn CR. Critical Nodes in Signalling Pathways: Insights Into Insulin Action. Nat Rev Mol Cell Biol (2006) 7(2):85–96. 10.1038/nrm1837 16493415

[22] Pugazhenthi S Nesterova A Sable C Heidenreich KA Boxer LM Heasley LE. Akt/Protein Kinase B Up-Regulates Bcl-2 Expression Through cAMP-response Element-Binding Protein. J Biol Chem (2000) 275(15):10761–6. 10.1074/jbc.275.15.10761 10753867

[23] Skolnik EY Batzer A Li N Lee CH Lowenstein E Mohammadi M. The Function of GRB2 in Linking the Insulin Receptor to Ras Signaling Pathways. Science (1993) 260(5116):1953–5. 10.1126/science.8316835 8316835

[24] Lavoie H Therrien M. Regulation of RAF Protein Kinases in ERK Signalling. Nat Rev Mol Cell Biol (2015) 16(5):281–98. 10.1038/nrm3979 25907612

[25] Kim SJ Kahn CR. Insulin Stimulates Phosphorylation of c-Jun, c-Fos, and Fos-related Proteins in Cultured Adipocytes. J Biol Chem (1994) 269(16):11887–92. 10.1016/S0021-9258(17)32656-X 7512956

[26] Dror E Dalmas E Meier DT Wueest S Thévenet J Thienel C. Postprandial Macrophage-Derived IL-1β Stimulates Insulin, and Both Synergistically Promote Glucose Disposal and Inflammation. Nat Immunol (2017) 18(3):283–92. 10.1038/ni.3659 28092375

[27] Maratou E Dimitriadis G Kollias A Boutati E Lambadiari V Mitrou P. Glucose Transporter Expression on the Plasma Membrane of Resting and Activated White Blood Cells. Eur J Clin Invest (2007) 37(4):282–90. 10.1111/j.1365-2362.2007.01786.x 17373964

[28] Yang P Wang X Wang D Shi Y Zhang M Yu T. Topical Insulin Application Accelerates Diabetic Wound Healing by Promoting Anti-Inflammatory Macrophage Polarization. J Cell Sci (2020) 133(19). 10.1242/jcs.235838 32878940

[29] Chen X Liu Y Zhang X. Topical Insulin Application Improves Healing by Regulating the Wound Inflammatory Response. Wound Repair Regen (2012) 20(3):425–34. 10.1111/j.1524-475X.2012.00792.x 22564234

[30] Yu T Gao M Yang P Pei Q Liu D Wang D. Topical Insulin Accelerates Cutaneous Wound Healing in Insulin-Resistant Diabetic Rats. Am J Transl Res (2017) 9(10):4682–93. PMC5666074 29118927

[31] Sun Q Li J Gao F. New Insights Into Insulin: The Anti-Inflammatory Effect and its Clinical Relevance. World J Diabetes (2014) 5(2):89–96. 10.4239/wjd.v5.i2.89 24765237 PMC3992527

[32] Maciver NJ Jacobs SR Wieman HL Wofford JA Coloff JL Rathmell JC. Glucose Metabolism in Lymphocytes is a Regulated Process With Significant Effects on Immune Cell Function and Survival. J Leukoc Biol (2008) 84(4):949–57. 10.1189/jlb.0108024 PMC2638731 18577716

[33] Kawahito S Kitahata H Oshita S. Problems Associated With Glucose Toxicity: Role of Hyperglycemia-Induced Oxidative Stress. World J Gastroenterol (2009) 15(33):4137–42. 10.3748/wjg.15.4137 PMC2738809 19725147

[34] Zhang Z Amorosa LF Coyle SM Macor MA Birnbaum MJ Lee LY. Insulin-Dependent Regulation of Mtorc2-Akt-Foxo Suppresses Tlr4 Signaling in Human Leukocytes: Relevance to Type 2 Diabetes. Diabetes (2016) 65(8):2224–34. 10.2337/db16-0027 27207509

[35] Tilich M Arora RR. Modulation of Toll-Like Receptors by Insulin. Am J Ther (2011) 18(5):e130–7. 10.1097/MJT.0b013e3181e71fa0 21326087

[36] Walrand S Guillet C Boirie Y Vasson MP. In Vivo Evidences That Insulin Regulates Human Polymorphonuclear Neutrophil Functions. J Leukoc Biol (2004) 76(6):1104–10. 10.1189/jlb.0104050 15345722

[37] Gerrits AJ Koekman CA Yildirim C Nieuwland R Akkerman JW. Insulin Inhibits Tissue Factor Expression in Monocytes. J Thromb Haemost (2009) 7(1):198–205. 10.1111/j.1538-7836.2008.03206.x 18983503

[38] Lu MC Yan ST Yin WY Koo M Lai NS. Risk of Rheumatoid Arthritis in Patients With Type 2 Diabetes: A Nationwide Population-Based Case-Control Study. PloS One (2014) 9(7):e101528. 10.1371/journal.pone.0101528 24988532 PMC4079714

[39] Fearon U Hanlon MM Wade SM Fletcher JM. Altered Metabolic Pathways Regulate Synovial Inflammation in Rheumatoid Arthritis. Clin Exp Immunol (2019) 197(2):170–80. 10.1111/cei.13228 PMC6642871 30357805

[40] Siddle K. Signalling by Insulin and IGF Receptors: Supporting Acts and New Players. J Mol Endocrinol (2011) 47(1):R1–10. 10.1530/JME-11-0022 21498522

[41] Verschure PJ van Marle J Joosten LA van den Berg WB. Chondrocyte IGF-1 Receptor Expression and Responsiveness to IGF-1 Stimulation in Mouse Articular Cartilage During Various Phases of Experimentally Induced Arthritis. Ann Rheum Dis (1995) 54(8):645–53. 10.1136/ard.54.8.645 PMC1009962 7677441

[42] Boström EA Svensson M Andersson S Jonsson IM Ekwall AK Eisler T. Resistin and Insulin/Insulin-Like Growth Factor Signaling in Rheumatoid Arthritis. Arthritis Rheum (2011) 63(10):2894–904. 10.1002/art.30527 21739426

[43] Lu MC Yu CL Chen HC Yu HC Huang HB Lai NS. Increased miR-223 Expression in T Cells From Patients With Rheumatoid Arthritis Leads to Decreased Insulin-Like Growth factor-1-mediated interleukin-10 Production. Clin Exp Immunol (2014) 177(3):641–51. 10.1111/cei.12374 PMC4137848 24816316

[44] Erlandsson MC Töyrä Silfverswärd S Nadali M Turkkila M Svensson MND Jonsson IM. Igf-1R Signalling Contributes to IL-6 Production and T Cell Dependent Inflammation in Rheumatoid Arthritis. Biochim Biophys Acta Mol Basis Dis (2017) 1863(9):2158–70. 10.1016/j.bbadis.2017.06.002 28583713

[45] Schäffler A Ehling A Neumann E Herfarth H Tarner I Schölmerich J. Adipocytokines in Synovial Fluid. Jama (2003) 290(13):1709–10. 10.1001/jama.290.13.1709-c 14519703

[46] Walrand S Guillet C Boirie Y Vasson MP. Insulin Differentially Regulates Monocyte and Polymorphonuclear Neutrophil Functions in Healthy Young and Elderly Humans. J Clin Endocrinol Metab (2006) 91(7):2738–48. 10.1210/jc.2005-1619 16621902

[47] Stentz FB Kitabchi AE. Activated T Lymphocytes in Type 2 Diabetes: Implications From In Vitro Studies. Curr Drug Targets (2003) 4(6):493–503. 10.2174/1389450033490966 12866664

[48] Viardot A Grey ST Mackay F Chisholm D. Potential Antiinflammatory Role of Insulin Via the Preferential Polarization of Effector T Cells Toward a T Helper 2 Phenotype. Endocrinology (2007) 148(1):346–53. 10.1210/en.2006-0686 17008395

[49] Helderman JH. Role of Insulin in the Intermediary Metabolism of the Activated Thymic-Derived Lymphocyte. J Clin Invest (1981) 67(6):1636–42. 10.1172/JCI110199 PMC370738 6787080

[50] Fischer HJ Sie C Schumann E Witte AK Dressel R van den Brandt J. The Insulin Receptor Plays a Critical Role in T Cell Function and Adaptive Immunity. J Immunol (2017) 198(5):1910–20. 10.4049/jimmunol.1601011 28115529

[51] Shin JY Kim SY Jeung MJ Eun SH Woo CW Yoon SY. Serum Adiponectin, C-reactive Protein and TNF-alpha Levels in Obese Korean Children. J Pediatr Endocrinol Metab (2008) 21(1):23–9. 10.1515/JPEM.2008.21.1.23 18404970

[52] Tao L Liu H Gong Y. Role and Mechanism of the Th17/Treg Cell Balance in the Development and Progression of Insulin Resistance. Mol Cell Biochem (2019) 459(1-2):183–8. 10.1007/s11010-019-03561-4 PMC6679830 31218568

[53] Vernal R Garcia-Sanz JA. Th17 and Treg Cells, Two New Lymphocyte Subpopulations With a Key Role in the Immune Response Against Infection. Infect Disord Drug Targets (2008) 8(4):207–20. 10.2174/187152608786734197 19075796

[54] Lee GR. The Balance of Th17 Versus Treg Cells in Autoimmunity. Int J Mol Sci (2018) 19(3):730. 10.3390/ijms19030730 PMC5877591 29510522

[55] Acuto O Michel F. CD28-Mediated Co-Stimulation: A Quantitative Support for TCR Signalling. Nat Rev Immunol (2003) 3(12):939–51. 10.1038/nri1248 14647476

[56] Chen L Flies DB. Molecular Mechanisms of T Cell Co-Stimulation and Co-Inhibition. Nat Rev Immunol (2013) 13(4):227–42. 10.1038/nri3405 PMC3786574 23470321

[57] Hwang JR Byeon Y Kim D Park SG. Recent Insights of T Cell Receptor-Mediated Signaling Pathways for T Cell Activation and Development. Exp Mol Med (2020) 52(5):750–61. 10.1038/s12276-020-0435-8 PMC7272404 32439954

[58] Zeng H Cohen S Guy C Shrestha S Neale G Brown SA. mTORC1 and Mtorc2 Kinase Signaling and Glucose Metabolism Drive Follicular Helper T Cell Differentiation. Immunity (2016) 45(3):540–54. 10.1016/j.immuni.2016.08.017 PMC5050556 27637146

[59] Zou X Wang S Zhang Y Wang X Yang W. The Role of Follicular T Helper Cells in the Onset and Treatment of Type 1 Diabetes. Int Immunopharmacol (2020) 84:106499. 10.1016/j.intimp.2020.106499 32298964

[60] Chi H. Regulation and Function of mTOR Signalling in T Cell Fate Decisions. Nat Rev Immunol (2012) 12(5):325–38. 10.1038/nri3198 PMC3417069 22517423

[61] Sun IH Oh MH Zhao L Patel CH Arwood ML Xu W. Mtor Complex 1 Signaling Regulates the Generation and Function of Central and Effector Foxp3(+) Regulatory T Cells. J Immunol (2018) 201(2):481–92. 10.4049/jimmunol.1701477 PMC6089237 29884702

[62] Chapman NM Zeng H Nguyen TM Wang Y Vogel P Dhungana Y. mTOR Coordinates Transcriptional Programs and Mitochondrial Metabolism of Activated T(reg) Subsets to Protect Tissue Homeostasis. Nat Commun (2018) 9(1):2095. 10.1038/s41467-018-04392-5 29844370 PMC5974344

[63] Mihaylova MM Shaw RJ. The AMPK Signalling Pathway Coordinates Cell Growth, Autophagy and Metabolism. Nat Cell Biol (2011) 13(9):1016–23. 10.1038/ncb2329 PMC3249400 21892142

[64] Tejera-Segura B López-Mejías R Domínguez-Luis MJ de Vera-González AM González-Delgado A Ubilla B. Incretins in Patients With Rheumatoid Arthritis. Arthritis Res Ther (2017) 19(1):229. 10.1186/s13075-017-1431-9 29041949 PMC5645916

[65] Hamada D Maynard R Schott E Drinkwater CJ Ketz JP Kates SL. Suppressive Effects of Insulin on Tumor Necrosis Factor-Dependent Early Osteoarthritic Changes Associated With Obesity and Type 2 Diabetes Mellitus. Arthritis Rheumatol (2016) 68(6):1392–402. 10.1002/art.39561 PMC4882234 26713606

[66] Qiao L Li Y Sun S. Insulin Exacerbates Inflammation in Fibroblast-Like Synoviocytes. Inflammation (2020) 43(3):916–36. 10.1007/s10753-020-01178-0 PMC7280329 31981062

[67] Lories RJ. Joint Homeostasis, Restoration, and Remodeling in Osteoarthritis. Best Pract Res Clin Rheumatol (2008) 22(2):209–20. 10.1016/j.berh.2007.12.001 18455680

[68] Pramojanee SN Phimphilai M Chattipakorn N Chattipakorn SC. Possible Roles of Insulin Signaling in Osteoblasts. Endocr Res (2014) 39(4):144–51. 10.3109/07435800.2013.879168 24679227

[69] Yang J Zhang X Wang W Liu J. Insulin Stimulates Osteoblast Proliferation and Differentiation Through ERK and PI3K in MG-63 Cells. Cell Biochem Funct (2010) 28(4):334–41. 10.1002/cbf.1668 20517899

[70] Zhang W Shen X Wan C Zhao Q Zhang L Zhou Q. Effects of Insulin and Insulin-Like Growth Factor 1 on Osteoblast Proliferation and Differentiation: Differential Signalling Via Akt and ERK. Cell Biochem Funct (2012) 30(4):297–302. 10.1002/cbf.2801 22249904

[71] Fulzele K DiGirolamo DJ Liu Z Xu J Messina JL Clemens TL. Disruption of the Insulin-Like Growth Factor Type 1 Receptor in Osteoblasts Enhances Insulin Signaling and Action. J Biol Chem (2007) 282(35):25649–58. 10.1074/jbc.M700651200 17553792

[72] Ghodsi M Larijani B Keshtkar AA Nasli-Esfahani E Alatab S Mohajeri-Tehrani MR. Mechanisms Involved in Altered Bone Metabolism in Diabetes: A Narrative Review. J Diabetes Metab Disord (2016) 15:52. 10.1186/s40200-016-0275-1 27891497 PMC5111345

[73] Ferron M Wei J Yoshizawa T Del Fattore A DePinho RA Teti A. Insulin Signaling in Osteoblasts Integrates Bone Remodeling and Energy Metabolism. Cell (2010) 142(2):296–308. 10.1016/j.cell.2010.06.003 20655470 PMC2910411

[74] Clemens TL Karsenty G. The Osteoblast: An Insulin Target Cell Controlling Glucose Homeostasis. J Bone Miner Res (2011) 26(4):677–80. 10.1002/jbmr.321 21433069

[75] Fulzele K Riddle RC DiGirolamo DJ Cao X Wan C Chen D. Insulin Receptor Signaling in Osteoblasts Regulates Postnatal Bone Acquisition and Body Composition. Cell (2010) 142(2):309–19. 10.1016/j.cell.2010.06.002 PMC2925155 20655471

[76] Xian L Wu X Pang L Lou M Rosen CJ Qiu T. Matrix IGF-1 Maintains Bone Mass by Activation of mTOR in Mesenchymal Stem Cells. Nat Med (2012) 18(7):1095–101. 10.1038/nm.2793 PMC3438316 22729283

[77] Fitter S Matthews MP Martin SK Xie J Ooi SS Walkley CR. Mtorc1 Plays an Important Role in Skeletal Development by Controlling Preosteoblast Differentiation. Mol Cell Biol (2017) 37(7):e00668-16. 10.1128/MCB.00668-16 28069737 PMC5359426

[78] Oh JH Lee NK. Up-Regulation of RANK Expression Via ERK1/2 by Insulin Contributes to the Enhancement of Osteoclast Differentiation. Mol Cells (2017) 40(5):371–7. 10.14348/molcells.2017.0025 PMC5463046 28535663

[79] Zhang Y Xu S Li K Tan K Liang K Wang J. Mtorc1 Inhibits Nf-κb/Nfatc1 Signaling and Prevents Osteoclast Precursor Differentiation, In Vitro and In Mice. J Bone Miner Res (2017) 32(9):1829–40. 10.1002/jbmr.3172 28520214

[80] Hiraiwa M Ozaki K Yamada T Iezaki T Park G Fukasawa K. Mtorc1 Activation in Osteoclasts Prevents Bone Loss in a Mouse Model of Osteoporosis. Front Pharmacol (2019) 10:684. 10.3389/fphar.2019.00684 31263418 PMC6585391

[81] Dai Q Xie F Han Y Ma X Zhou S Jiang L. Inactivation of Regulatory-associated Protein of mTOR (Raptor)/Mammalian Target of Rapamycin Complex 1 (Mtorc1) Signaling in Osteoclasts Increases Bone Mass by Inhibiting Osteoclast Differentiation in Mice. J Biol Chem (2017) 292(1):196–204. 10.1074/jbc.M116.764761 27879318 PMC5217679

[82] Courties A Sellam J Berenbaum F. Metabolic Syndrome-Associated Osteoarthritis. Curr Opin Rheumatol (2017) 29(2):214–22. 10.1097/BOR.0000000000000373 28072592

[83] Veronese N Cooper C Reginster JY Hochberg M Branco J Bruyère O. Type 2 Diabetes Mellitus and Osteoarthritis. Semin Arthritis Rheum (2019) 49(1):9–19. 10.1016/j.semarthrit.2019.01.005 30712918 PMC6642878

[84] Ribeiro M López de Figueroa P Blanco FJ Mendes AF Caramés B. Insulin Decreases Autophagy and Leads to Cartilage Degradation. Osteoarthritis Cartilage (2016) 24(4):731–9. 10.1016/j.joca.2015.10.017 26549531

[85] Zheng L Zhang Z Sheng P Mobasheri A. The Role of Metabolism in Chondrocyte Dysfunction and the Progression of Osteoarthritis. Ageing Res Rev (2020) 66:101249. 10.1016/j.arr.2020.101249 33383189

[86] Zhang Y Vasheghani F Li YH Blati M Simeone K Fahmi H. Cartilage-Specific Deletion of mTOR Upregulates Autophagy and Protects Mice From Osteoarthritis. Ann Rheum Dis (2015) 74(7):1432–40. 10.1136/annrheumdis-2013-204599 24651621

[87] Ieronymaki E Daskalaki MG Lyroni K Tsatsanis C. Insulin Signaling and Insulin Resistance Facilitate Trained Immunity in Macrophages Through Metabolic and Epigenetic Changes. Front Immunol (2019) 10:1330. 10.3389/fimmu.2019.01330 31244863 PMC6581697

[88] Klauder J Henkel J Vahrenbrink M Wohlenberg AS Camargo RG Püschel GP. Direct and Indirect Modulation of LPS-induced Cytokine Production by Insulin in Human Macrophages. Cytokine (2020) 136:155241. 10.1016/j.cyto.2020.155241 32799102

[89] Svenson KL Pollare T Lithell H Hällgren R. Impaired Glucose Handling in Active Rheumatoid Arthritis: Relationship to Peripheral Insulin Resistance. Metabolism (1988) 37(2):125–30. 10.1016/S0026-0495(98)90005-1 2893241

[90] Dessein PH Joffe BI. Insulin Resistance and Impaired Beta Cell Function in Rheumatoid Arthritis. Arthritis Rheumatol (2006) 54(9):2765–75. 10.1002/art.22053 16947779

[91] Dessein PH Stanwix AE Joffe BI. Cardiovascular Risk in Rheumatoid Arthritis Versus Osteoarthritis: Acute Phase Response Related Decreased Insulin Sensitivity and High-Density Lipoprotein Cholesterol as Well as Clustering of Metabolic Syndrome Features in Rheumatoid Arthritis. Arthritis Res (2002) 4(5):R5. 10.1186/ar428 12223108 PMC125299

[92] Rosenvinge A Krogh-Madsen R Baslund B Pedersen BK. Insulin Resistance in Patients With Rheumatoid Arthritis: Effect of anti-TNFalpha Therapy. Scand J Rheumatol (2007) 36(2):91–6. 10.1080/03009740601179605 17476613

[93] Tejera-Segura B López-Mejías R de Vera-González AM Jiménez-Sosa A Olmos JM Hernández JL. Relationship Between Insulin Sensitivity and β-Cell Secretion in Nondiabetic Subjects With Rheumatoid Arthritis. J Rheumatol (2019) 46(3):229–36. 10.3899/jrheum.180198 30275261

[94] Shahin D Eltoraby E Mesbah A Houssen M. Insulin Resistance in Early Untreated Rheumatoid Arthritis Patients. Clin Biochem (2010) 43(7-8):661–5. 10.1016/j.clinbiochem.2010.01.012 20144599

[95] Ferraz-Amaro I García-Dopico JA Medina-Vega L González-Gay MA Díaz-González F. Impaired Beta Cell Function is Present in Nondiabetic Rheumatoid Arthritis Patients. Arthritis Res Ther (2013) 15(1):R17. 10.1186/ar4149 23339356 PMC3672807

[96] Tchetina EV Markova GA Sharapova EP. Insulin Resistance in Osteoarthritis: Similar Mechanisms to Type 2 Diabetes Mellitus. J Nutr Metab (2020) 2020:4143802. 10.1155/2020/4143802 32566279 PMC7261331

[97] Scheen AJ Esser N Paquot N. Antidiabetic Agents: Potential Anti-Inflammatory Activity Beyond Glucose Control. Diabetes Metab (2015) 41(3):183–94. 10.1016/j.diabet.2015.02.003 25794703

[98] Ursini F Russo E Pellino G D’Angelo S Chiaravalloti A De Sarro G. Metformin and Autoimmunity: A “New Deal” of an Old Drug. Front Immunol (2018) 9:1236. 10.3389/fimmu.2018.01236 29915588 PMC5994909

[99] Ursini F Grembiale RD D’Antona L Gallo E D’Angelo S Citraro R. Oral Metformin Ameliorates Bleomycin-Induced Skin Fibrosis. J Invest Dermatol (2016) 136(9):1892–4. 10.1016/j.jid.2016.05.097 27251791

[100] Salvatore T Pafundi PC Galiero R Gjeloshi K Masini F Acierno C. Metformin: A Potential Therapeutic Tool for Rheumatologists. Pharmaceuticals (Basel) (2020) 13(9):234. 10.3390/ph13090234 PMC7560003 32899806

[101] Owen MR Doran E Halestrap AP. Evidence That Metformin Exerts its Anti-Diabetic Effects Through Inhibition of Complex 1 of the Mitochondrial Respiratory Chain. Biochem J (2000) 348 Pt 3(Pt 3):607–14. 10.1042/bj3480607 PMC1221104 10839993

[102] Ota S Horigome K Ishii T Nakai M Hayashi K Kawamura T. Metformin Suppresses glucose-6-phosphatase Expression by a Complex I Inhibition and AMPK Activation-Independent Mechanism. Biochem Biophys Res Commun (2009) 388(2):311–6. 10.1016/j.bbrc.2009.07.164 19664596

[103] Kelly B Tannahill GM Murphy MP O’Neill LA. Metformin Inhibits the Production of Reactive Oxygen Species From NADH:Ubiquinone Oxidoreductase to Limit Induction of Interleukin-1β (Il-1β) and Boosts Interleukin-10 (Il-10) in Lipopolysaccharide (LPS)-Activated Macrophages. J Biol Chem (2015) 290(33):20348–59. 10.1074/jbc.M115.662114 PMC4536441 26152715

[104] Kang KY Kim YK Yi H Kim J Jung HR Kim IJ. Metformin Downregulates Th17 Cells Differentiation and Attenuates Murine Autoimmune Arthritis. Int Immunopharmacol (2013) 16(1):85–92. 10.1016/j.intimp.2013.03.020 23557965

[105] Gallagher L Cregan S Biniecka M Cunningham C Veale DJ Kane DJ. Insulin-Resistant Pathways are Associated With Disease Activity in Rheumatoid Arthritis and Are Subject to Disease Modification Through Metabolic Reprogramming: A Potential Novel Therapeutic Approach. Arthritis Rheumatol (2020) 72(6):896–902. 10.1002/art.41190 31840936

[106] Coughlan KA Valentine RJ Ruderman NB Saha AK. AMPK Activation: A Therapeutic Target for Type 2 Diabetes? Diabetes Metab Syndr Obes (2014) 7:241–53. 10.2147/DMSO.S43731 PMC4075959 25018645

[107] Indo Y Takeshita S Ishii KA Hoshii T Aburatani H Hirao A. Metabolic Regulation of Osteoclast Differentiation and Function. J Bone Miner Res (2013) 28(11):2392–9. 10.1002/jbmr.1976 23661628

[108] Yan H Zhou HF Hu Y Pham CT. Suppression of Experimental Arthritis Through AMP-activated Protein Kinase Activation and Autophagy Modulation. J Rheum Dis Treat (2015) 1(1):5. 10.23937/2469-5726/1510005 26120598 PMC4479345

[109] Shiojiri T Wada K Nakajima A Katayama K Shibuya A Kudo C. PPAR Gamma Ligands Inhibit Nitrotyrosine Formation and Inflammatory Mediator Expressions in Adjuvant-Induced Rheumatoid Arthritis Mice. Eur J Pharmacol (2002) 448(2-3):231–8. 10.1016/S0014-2999(02)01946-5 12144946

[110] Meier CA Chicheportiche R Juge-Aubry CE Dreyer MG Dayer JM. Regulation of the Interleukin-1 Receptor Antagonist in THP-1 Cells by Ligands of the Peroxisome Proliferator-Activated Receptor Gamma. Cytokine (2002) 18(6):320–8. 10.1006/cyto.2002.1945 12160520

[111] Abdelrahman M Sivarajah A Thiemermann C. Beneficial Effects of PPAR-gamma Ligands in Ischemia-Reperfusion Injury, Inflammation and Shock. Cardiovasc Res (2005) 65(4):772–81. 10.1016/j.cardiores.2004.12.008 15721857

[112] Hanefeld M Marx N Pfützner A Baurecht W Lübben G Karagiannis E. Anti-Inflammatory Effects of Pioglitazone and/or Simvastatin in High Cardiovascular Risk Patients With Elevated High Sensitivity C-reactive Protein: The PIOSTAT Study. J Am Coll Cardiol (2007) 49(3):290–7. 10.1016/j.jacc.2006.08.054 17239709

[113] Radenković M. Pioglitazone and Endothelial Dysfunction: Pleiotropic Effects and Possible Therapeutic Implications. Sci Pharm (2014) 82(4):709–21. 10.3797/scipharm.1407-16 PMC4500538 26171320

[114] Giannini S Serio M Galli A. Pleiotropic Effects of Thiazolidinediones: Taking a Look Beyond Antidiabetic Activity. J Endocrinol Invest (2004) 27(10):982–91. 10.1007/BF03347546 15762051

[115] Remels AH Langen RC Gosker HR Russell AP Spaapen F Voncken JW. Ppargamma Inhibits NF-kappaB-dependent Transcriptional Activation in Skeletal Muscle. Am J Physiol Endocrinol Metab (2009) 297(1):E174–83. 10.1152/ajpendo.90632.2008 19417127

[116] Hou Y Moreau F Chadee K. Pparγ is an E3 Ligase That Induces the Degradation of Nfκb/P65. Nat Commun (2012) 3:1300. 10.1038/ncomms2270 23250430

[117] Murakami-Nishida S Matsumura T Senokuchi T Ishii N Kinoshita H Yamada S. Pioglitazone Suppresses Macrophage Proliferation in Apolipoprotein-E Deficient Mice by Activating Pparγ. Atherosclerosis (2019) 286:30–9. 10.1016/j.atherosclerosis.2019.04.229 31096071

[118] Yano M Matsumura T Senokuchi T Ishii N Motoshima H Taguchi T. Troglitazone Inhibits Oxidized Low-Density Lipoprotein-Induced Macrophage Proliferation: Impact of the Suppression of Nuclear Translocation of ERK1/2. Atherosclerosis (2007) 191(1):22–32. 10.1016/j.atherosclerosis.2006.04.022 16725145

[119] Chinetti G Griglio S Antonucci M Torra IP Delerive P Majd Z. Activation of Proliferator-Activated Receptors Alpha and Gamma Induces Apoptosis of Human Monocyte-Derived Macrophages. J Biol Chem (1998) 273(40):25573–80. 10.1074/jbc.273.40.25573 9748221

[120] Liu Y Qu Y Liu L Zhao H Ma H Si M. Ppar-γ Agonist Pioglitazone Protects Against IL-17 Induced Intervertebral Disc Inflammation and Degeneration Via Suppression of NF-κb Signaling Pathway. Int Immunopharmacol (2019) 72:138–47. 10.1016/j.intimp.2019.04.012 30981079

[121] Tsubouchi Y Kawahito Y Kohno M Inoue K Hla T Sano H. Feedback Control of the Arachidonate Cascade in Rheumatoid Synoviocytes by 15-deoxy-Delta(12,14)-prostaglandin J2. Biochem Biophys Res Commun (2001) 283(4):750–5. 10.1006/bbrc.2001.4847 11350047

[122] Cuzzocrea S Mazzon E Dugo L Patel NS Serraino I Di Paola R. Reduction in the Evolution of Murine Type II Collagen-Induced Arthritis by Treatment With Rosiglitazone, a Ligand of the Peroxisome Proliferator-Activated Receptor Gamma. Arthritis Rheum (2003) 48(12):3544–56. 10.1002/art.11351 14674008

[123] Kawahito Y Kondo M Tsubouchi Y Hashiramoto A Bishop-Bailey D Inoue K. 15-Deoxy-Delta(12,14)-PGJ(2) Induces Synoviocyte Apoptosis and Suppresses Adjuvant-Induced Arthritis in Rats. J Clin Invest (2000) 106(2):189–97. 10.1172/JCI9652 PMC314310 10903334

[124] Shahin D Toraby EE Abdel-Malek H Boshra V Elsamanoudy AZ Shaheen D. Effect of Peroxisome Proliferator-Activated Receptor Gamma Agonist (Pioglitazone) and Methotrexate on Disease Activity in Rheumatoid Arthritis (Experimental and Clinical Study). Clin Med Insights Arthritis Musculoskelet Disord (2011) 4:1–10. 10.4137/CMAMD.S5951 21339857 PMC3040074

[125] Ormseth MJ Oeser AM Cunningham A Bian A Shintani A Solus J. Peroxisome Proliferator-Activated Receptor γ Agonist Effect on Rheumatoid Arthritis: A Randomized Controlled Trial. Arthritis Res Ther (2013) 15(5):R110. 10.1186/ar4290 24020899 PMC3978636

[126] Tomita T Kakiuchi Y Tsao PS. THR0921, a Novel Peroxisome Proliferator-Activated Receptor Gamma Agonist, Reduces the Severity of Collagen-Induced Arthritis. Arthritis Res Ther (2006) 8(1):R7. 10.1186/ar1856 16356194 PMC1526548

[127] Koufany M Moulin D Bianchi A Muresan M Sebillaud S Netter P. Anti-Inflammatory Effect of Antidiabetic Thiazolidinediones Prevents Bone Resorption Rather Than Cartilage Changes in Experimental Polyarthritis. Arthritis Res Ther (2008) 10(1):R6. 10.1186/ar2354 18199331 PMC2374462

[128] Fahmi H Di Battista JA Pelletier JP Mineau F Ranger P Martel-Pelletier J. Peroxisome Proliferator–Activated Receptor Gamma Activators Inhibit interleukin-1beta-induced Nitric Oxide and Matrix Metalloproteinase 13 Production in Human Chondrocytes. Arthritis Rheumatol (2001) 44(3):595–607. 10.1002/1529-0131(200103)44:3<595::AID-ANR108>3.0.CO;2-8 11263774

[129] Inoue H Tanabe T Umesono K. Feedback Control of Cyclooxygenase-2 Expression Through Ppargamma. J Biol Chem (2000) 275(36):28028–32. 10.1074/jbc.M001387200 10827178

[130] Fahmi H Pelletier JP Martel-Pelletier J. Ppargamma Ligands as Modulators of Inflammatory and Catabolic Responses in Arthritis. An Overview. J Rheumatol (2002) 29(1):3–14. 11824967

[131] Yazbeck R Howarth GS Abbott CA. Dipeptidyl Peptidase Inhibitors, an Emerging Drug Class for Inflammatory Disease? Trends Pharmacol Sci (2009) 30(11):600–7. 10.1016/j.tips.2009.08.003 19837468

[132] Ohnuma K Hosono O Dang NH Morimoto C. Dipeptidyl Peptidase in Autoimmune Pathophysiology. Adv Clin Chem (2011) 53:51–84. 10.1016/B978-0-12-385855-9.00003-5 21404914

[133] Seong JM Yee J Gwak HS. Dipeptidyl Peptidase-4 Inhibitors Lower the Risk of Autoimmune Disease in Patients With Type 2 Diabetes Mellitus: A Nationwide Population-Based Cohort Study. Br J Clin Pharmacol (2019) 85(8):1719–27. 10.1111/bcp.13955 PMC6624390 30964554

[134] Kim SC Schneeweiss S Glynn RJ Doherty M Goldfine AB Solomon DH. Dipeptidyl Peptidase-4 Inhibitors in Type 2 Diabetes may Reduce the Risk of Autoimmune Diseases: A Population-Based Cohort Study. Ann Rheum Dis (2015) 74(11):1968–75. 10.1136/annrheumdis-2014-205216 PMC4263684 24919467

[135] Tanaka S Murakami T Nonaka N Ohnuki T Yamada M Sugita T. Anti-Arthritic Effects of the Novel Dipeptidyl Peptidase IV Inhibitors TMC-2A and TSL-225. Immunopharmacology (1998) 40(1):21–6. 10.1016/S0162-3109(98)00014-9 9776475

[136] Tanaka S Murakami T Horikawa H Sugiura M Kawashima K Sugita T. Suppression of Arthritis by the Inhibitors of Dipeptidyl Peptidase IV. Int J Immunopharmacol (1997) 19(1):15–24. 10.1016/S0192-0561(97)00004-0 9226475

[137] Ibrahim SSA Salama MA Selima E Shehata RR. Sitagliptin and Tofacitinib Ameliorate Adjuvant Induced Arthritis Via Modulating the Cross Talk Between JAK/STAT and TLR-4/NF-κb Signaling Pathways. Life Sci (2020) 260:118261. 10.1016/j.lfs.2020.118261 32795539

[138] Hu N Gong X Yin S Li Q Chen H Li Y. Saxagliptin Suppresses Degradation of Type II Collagen and Aggrecan in Primary Human Chondrocytes: A Therapeutic Implication in Osteoarthritis. Artif Cells Nanomed Biotechnol (2019) 47(1):3239–45. 10.1080/21691401.2019.1647223 31364869

[139] Müller TD Finan B Bloom SR D’Alessio D Drucker DJ Flatt PR. Glucagon-Like Peptide 1 (GLP-1). Mol Metab (2019) 30:72–130. 10.1016/j.molmet.2019.09.010 31767182 PMC6812410

[140] Chen CY Tsai CY. From Endocrine to Rheumatism: do Gut Hormones Play Roles in Rheumatoid Arthritis? Rheumatol (Oxford) (2014) 53(2):205–12. 10.1093/rheumatology/ket255 23882111

[141] Du X Zhang H Zhang W Wang Q Wang W Ge G. The Protective Effects of Lixisenatide Against Inflammatory Response in Human Rheumatoid Arthritis Fibroblast-Like Synoviocytes. Int Immunopharmacol (2019) 75:105732. 10.1016/j.intimp.2019.105732 31336333

[142] Chen J Xie JJ Shi KS Gu YT Wu CC Xuan J. Glucagon-Like Peptide-1 Receptor Regulates Endoplasmic Reticulum Stress-Induced Apoptosis and the Associated Inflammatory Response in Chondrocytes and the Progression of Osteoarthritis in Rat. Cell Death Dis (2018) 9(2):212. 10.1038/s41419-017-0217-y 29434185 PMC5833344

[143] Tao Y Ge G Wang Q Wang W Zhang W Bai J. Exenatide Ameliorates Inflammatory Response in Human Rheumatoid Arthritis Fibroblast-Like Synoviocytes. IUBMB Life (2019) 71(7):969–77. 10.1002/iub.2031 30897288

[144] Zheng W Pan H Wei L Gao F Lin X. Dulaglutide Mitigates Inflammatory Response in Fibroblast-Like Synoviocytes. Int Immunopharmacol (2019) 74:105649. 10.1016/j.intimp.2019.05.034 31185450

[145] Li H Chen J Li B Fang X. The Protective Effects of Dulaglutide Against Advanced Glycation End Products (Ages)-Induced Degradation of Type II Collagen and Aggrecan in Human SW1353 Chondrocytes. Chem Biol Interact (2020) 322:108968. 10.1016/j.cbi.2020.108968 32004530

[146] Hoes JN van der Goes MC van Raalte DH van der Zijl NJ den Uyl D Lems WF. Glucose Tolerance, Insulin Sensitivity and β-Cell Function in Patients With Rheumatoid Arthritis Treated With or Without Low-to-Medium Dose Glucocorticoids. Ann Rheum Dis (2011) 70(11):1887–94. 10.1136/ard.2011.151464 21908880

[147] Giles JT Danielides S Szklo M Post WS Blumenthal RS Petri M. Insulin Resistance in Rheumatoid Arthritis: Disease-Related Indicators and Associations With the Presence and Progression of Subclinical Atherosclerosis. Arthritis Rheumatol (2015) 67(3):626–36. 10.1002/art.38986 PMC6058692 25504899

[148] Tilg H Moschen AR. Inflammatory Mechanisms in the Regulation of Insulin Resistance. Mol Med (2008) 14(3-4):222–31. 10.2119/2007-00119.Tilg PMC2215762 18235842

[149] Giles JT Allison M Blumenthal RS Post W Gelber AC Petri M. Abdominal Adiposity in Rheumatoid Arthritis: Association With Cardiometabolic Risk Factors and Disease Characteristics. Arthritis Rheum (2010) 62(11):3173–82. 10.1002/art.27629 PMC2962724 20589684

[150] Burska AN Sakthiswary R Sattar N. Effects of Tumour Necrosis Factor Antagonists on Insulin Sensitivity/Resistance in Rheumatoid Arthritis: A Systematic Review and Meta-Analysis. PloS One (2015) 10(6):e0128889. 10.1371/journal.pone.0128889 26110878 PMC4482317

[151] Hotamisligil GS Peraldi P Budavari A Ellis R White MF Spiegelman BM. Irs-1-mediated Inhibition of Insulin Receptor Tyrosine Kinase Activity in TNF-alpha- and Obesity-Induced Insulin Resistance. Science (1996) 271(5249):665–8. 10.1126/science.271.5249.665 8571133

[152] Kanety H Feinstein R Papa MZ Hemi R Karasik A. Tumor Necrosis Factor Alpha-Induced Phosphorylation of Insulin Receptor Substrate-1 (IRS-1). Possible Mechanism for Suppression of Insulin-Stimulated Tyrosine Phosphorylation of IRS-1. J Biol Chem (1995) 270(40):23780–4. 10.1074/jbc.270.40.23780 7559552

[153] Gonzalez-Gay MA Gonzalez-Juanatey C Vazquez-Rodriguez TR Miranda-Filloy JA Llorca J. Insulin Resistance in Rheumatoid Arthritis: The Impact of the anti-TNF-alpha Therapy. Ann N Y Acad Sci (2010) 1193:153–9. 10.1111/j.1749-6632.2009.05287.x 20398022

[154] Gonzalez-Gay MA De Matias JM Gonzalez-Juanatey C Garcia-Porrua C Sanchez-Andrade A Martin J. Anti-Tumor Necrosis Factor-Alpha Blockade Improves Insulin Resistance in Patients With Rheumatoid Arthritis. Clin Exp Rheumatol (2006) 24(1):83–6. 16539824

[155] Antohe JL Bili A Sartorius JA Kirchner HL Morris SJ Dancea S. Diabetes Mellitus Risk in Rheumatoid Arthritis: Reduced Incidence With Anti-Tumor Necrosis Factor α Therapy. Arthritis Care Res (Hoboken) (2012) 64(2):215–21. 10.1002/acr.20657 21972198

[156] Wasko MC Kay J Hsia EC Rahman MU. Diabetes Mellitus and Insulin Resistance in Patients With Rheumatoid Arthritis: Risk Reduction in a Chronic Inflammatory Disease. Arthritis Care Res (Hoboken) (2011) 63(4):512–21. 10.1002/acr.20414 21452264

[157] Stagakis I Bertsias G Karvounaris S Kavousanaki M Virla D Raptopoulou A. Anti-Tumor Necrosis Factor Therapy Improves Insulin Resistance, Beta Cell Function and Insulin Signaling in Active Rheumatoid Arthritis Patients With High Insulin Resistance. Arthritis Res Ther (2012) 14(3):R141. 10.1186/ar3874 22691241 PMC3446524

[158] Ogata A Morishima A Hirano T Hishitani Y Hagihara K Shima Y. Improvement of HbA1c During Treatment With Humanised Anti-Interleukin 6 Receptor Antibody, Tocilizumab. Ann Rheum Dis (2011) 70(6):1164–5. 10.1136/ard.2010.132845 20980285

[159] Schultz O Oberhauser F Saech J Rubbert-Roth A Hahn M Krone W. Effects of Inhibition of Interleukin-6 Signalling on Insulin Sensitivity and Lipoprotein (a) Levels in Human Subjects With Rheumatoid Diseases. PloS One (2010) 5(12):e14328. 10.1371/journal.pone.0014328 21179199 PMC3001451

[160] Castañeda S Remuzgo-Martínez S López-Mejías R Genre F Calvo-Alén J Llorente I. Rapid Beneficial Effect of the IL-6 Receptor Blockade on Insulin Resistance and Insulin Sensitivity in non-Diabetic Patients With Rheumatoid Arthritis. Clin Exp Rheumatol (2019) 37(3):465–73. 30418124

[161] van Asseldonk EJ van Poppel PC Ballak DB Stienstra R Netea MG Tack CJ. One Week Treatment With the IL-1 Receptor Antagonist Anakinra Leads to a Sustained Improvement in Insulin Sensitivity in Insulin Resistant Patients With Type 1 Diabetes Mellitus. Clin Immunol (2015) 160(2):155–62. 10.1016/j.clim.2015.06.003 26073226

[162] Ruscitti P Masedu F Alvaro S Airò P Battafarano N Cantarini L. Anti-Interleukin-1 Treatment in Patients With Rheumatoid Arthritis and Type 2 Diabetes (TRACK): A Multicentre, Open-Label, Randomised Controlled Trial. PloS Med (2019) 16(9):e1002901. 10.1371/journal.pmed.1002901 31513665 PMC6742232

[163] Ursini F Russo E Letizia Hribal M Mauro D Savarino F Bruno C. Abatacept Improves Whole-Body Insulin Sensitivity in Rheumatoid Arthritis: An Observational Study. Med (Baltimore) (2015) 94(21):e888. 10.1097/MD.0000000000000888 PMC4616417 26020396

